# Long chain saturated and unsaturated fatty acids exert opposing effects on viability and function of GLP-1-producing cells: Mechanisms of lipotoxicity

**DOI:** 10.1371/journal.pone.0177605

**Published:** 2017-05-16

**Authors:** Ketan Thombare, Stelia Ntika, Xuan Wang, Camilla Krizhanovskii

**Affiliations:** 1 Södertälje Hospital, Department of Internal Medicine, Södertälje, Sweden; 2 Karolinska Institute, Department of Molecular Medicine and Surgery, Stockholm, Sweden; 3 Uppsala University, Department of Medical Cell Biology, Uppsala, Sweden; University of Wisconsin Madison, UNITED STATES

## Abstract

**Background and aim:**

Fatty acids acutely stimulate GLP-1 secretion from L-cells *in vivo*. However, a high fat diet has been shown to reduce the density of L-cells in the mouse intestine and a positive correlation has been indicated between L-cell number and GLP-1 secretion. Thus, the mechanism of fatty acid-stimulated GLP-1 secretion, potential effects of long-term exposure to elevated levels of different fatty acid species, and underlying mechanisms are not fully understood. In the present study, we sought to determine how long-term exposure to saturated (16:0) and unsaturated (18:1) fatty acids, by direct effects on GLP-1-producing cells, alter function and viability, and the underlying mechanisms.

**Methods:**

GLP-1-secreting GLUTag cells were cultured in the presence/absence of saturated (16:0) and unsaturated (18:1) fatty acids (0.125 mM for 48 h, followed by analyses of viability and apoptosis, as well as involvement of fatty acid oxidation, free fatty acid receptors (FFAR1) and ceramide synthesis. In addition, effects on the expression of proglucagon, prohormone convertase 1/3 (PC1/3), free fatty acid receptors (FFAR1, FFAR3), sodium glucose co-transporter (SGLT) and subsequent secretory response were determined.

**Results:**

Saturated (16:0) and unsaturated (18:1) fatty acids exerted opposing effects on the induction of apoptosis (1.4-fold increase in DNA fragmentation by palmitate and a 0.5-fold reduction by oleate; p<0.01). Palmitate-induced apoptosis was associated with increased ceramide content and co-incubation with Fumonisin B1 abolished this lipo apoptosis. Oleate, on the other hand, reduced ceramide content, and—unlike palmitate—upregulated FFAR1 and FFAR3, evoking a 2-fold increase in FFAR1-mediated GLP-1 secretion following acute exposure to 0.125 mmol/L palmitate; (p<0.05).

**Conclusion/Interpretation:**

Saturated (16:0), but not unsaturated (18:1), fatty acids induce ceramide-mediated apoptosis of GLP-1-producing cells. Further, unsaturated fatty acids confer lipoprotection, enhancing viability and function of GLP-1-secreting cells. These data provide potential mechanistic insight contributing to reduced L-cell mass following a high fat diet and differential effects of saturated and unsaturated fatty acids on GLP-1 secretion *in vivo*.

## Introduction

Glucagon-like peptide-1 (GLP-1) has a central role in type 2 diabetes (T2D) due to its potentiating effects on insulin secretion and the successful use of GLP-1 analogs in T2D therapy. GLP-1 is synthesized from the preproglucagon gene (*Gcg*) in a subset of enteroendocrine cells (EECs), denominated L-cells. The L-cells are found scattered in the intestinal epithelium with increasing numbers towards the distal ileum and colon, representing in totality < 1% of the epithelial cells. L-cells are polarized, exhibiting an apical surface with microvilli in contact with the lumen, and a basolateral surface from which secretory vesicles exocytose [1]. GLP-1 secretion is stimulated by nutrient intake (carbohydrates, proteins, and fats) and potentiates glucose-stimulated insulin secretion (GSIS)–*i*.*e*. the incretin effect. In addition to its insulinotropic effects, GLP-1 also stimulates β-cell proliferative and anti-apoptotic pathways, exerts protective effects on cardiomyocytes, reduces insulin resistance, while also inhibiting glucagon release, gastric emptying, and food intake [2].

Reduced plasma GLP-1 levels have been observed in T2D, but also with increased BMI and obesity independent of T2D [3, 4]. Continuous administration of GLP-1 to T2D patients restores GSIS and normalizes glycemia [2]. However, due to rapid degradation by Dipeptidyl peptidase– 4 (DPP-4), stable analogs of GLP-1 such as exendin-4 and liraglutide, as well as DPP-4 inhibitors such as sitagliptin, have been developed for T2D therapy.

Today’s incretin therapy has achieved great success and arguably constitutes the best available pharmaceutical intervention for the treatment of T2D. However, enhancing endogenous GLP-1 production is a novel avenue of incretin therapy, and would avoid potential deleterious effects of long term treatment with GLP-1 agonists on beta cell function [5], and offer many advantages to current incretin therapy. *I*.*e*. GLP-1 would be released by its native route directly into the portal vein—where regulatory GLP-1-sensitive glucose sensors are expressed—prior to hepatic passage [6]. Further, the pulsatile nature of endogenous GLP-1 secretion would be maintained [7], which may prevent GLP-1 resistance [8].

Potentiating endogenous incretin secretion requires detailed knowledge and understanding of the regulation of GLP-1-producing L-cells. Increased BMI and obesity are characterized by increased levels of circulating free fatty acids and hyperlipidemia. Studies on the effects of lipids and fatty acids on the function of GLP-1-secreting cells reveal that generation of long-chain fatty acids greater than C10 is a critical step for fat-induced stimulation of GLP-1 secretion in humans [9, 10]. Further, differential effects and mechanisms have been identified for stimulation by different fatty acid species, where vascular *vs*. luminal exposure is also indicated to be of importance [11].

Recently, focus has also been given to the possible alteration of L-cell mass in diabetic subjects and its implication for the endogenous incretin response. Reports have indicated that L-cell mass can be regulated by external stimuli [12, 13]. Short chain fatty acids have been indicated to increase L-cell mass [14], and a high fat diet (HFD) has been shown to reduce the density of L-cells in the distal parts of the mouse intestine [15, 16], where differential direct effects of different fatty acid species on the viability of GLP-1-producing cells have been reported [17], but the mechanisms remain unknown. Importantly, an increased L-cell number has also been associated with increased GLP-1 secretion [18]. Collectively these data support the possibility of modulating L-cell density to enhance endogenous GLP-1 secretion.

The association of reduced L-cell mass with obesity, insulin resistance, and defective incretin response, provokes questions as to how fatty acids can modulate not only the function but also the viability of L-cells. Non-adipose tissue accumulation of triglycerides (steatosis) is an indicator of lipid overload and hyperlipidemia in humans and animal models, and associated with impaired insulin signaling and lipotoxicity [19]. Triglyceride accumulation in tissue and triglycerides themselves are most likely inert in terms of lipotoxicity [20, 21]. However, non-adipose tissue has a limited capacity for triglyceride storage and fatty acids channeled to other metabolic fates, such as production of reactive oxygen species (ROS) or ceramide, are indicated to mediate the lipotoxic effects. Carnitine acyltransferase I (CPT-1) mediated mitochondrial fatty acid uptake enables fatty acid oxidation generating ROS as a natural byproduct. Hyperlipidemia and increased fatty acid oxidation may increase ROS to dangerous levels with detrimental effects on cell survival and function. Also the generation of lipid metabolites such as ceramide, composed of sphingosine and a fatty acid, resulting from breakdown of sphingomyelin and complex sphingolipids, or through *de novo* synthesis., have been shown to be mediating lipotoxic effects in some cell types. In addition to this, GLP-1 secreting cells express fatty acid receptors like FFAR1 indicated to be involved in the secretory response of GLP-1 secreting cells in response to fatty acids. However, the more long term effects of continuous activation of FFAR1 following a high fat diet and hyperlipidemia is not well understood.

Recent research has elucidated many aspects of fatty acid-stimulated secretion, but more detailed knowledge is needed, especially with regards to the effects of long term exposure to fatty acids in terms of viability as well as function. Consequently, we sought to determine the mechanisms mediating lipoapoptosis of GLP-1-producing cells and the roles of different fatty acid species in viability and function, aiming to further elucidate the molecular regulation of GLP-1-producing cells in health and disease.

## Materials and methods

### Cell culture and in vitro exposure

As a model of enteroendocrine L-cells, GLUTag cells—an immortalized murine enteroendocrine cell line expressing the proglucagon gene and secreting the glucagon-like peptides [22]—were used. GLUTag cells recapitulate the response of primary intestinal L-cells to physiological and pharmacological GLP-1 secretagogues [23, 24] and constitute one of the best models of the L-cell.

The GLP-1-secreting GLUTag cell line (source: glucagon-producing enteroendocrine cell tumor that arose in transgenic mice generated on an out-bred CD-1 background) [24], graciously donated by Dr. Neil Portwood at Karolinska Institutet, Solna, Sweden, and originally from Dr. Daniel J. Drucker, Mount Sinai Hospital, Samuel Lunenfeld Research Institute, University of Toronto, Canada, was cultured in DMEM (Thermo Fisher Scientific, Waltham, MA) supplemented with 10% fetal bovine serum (FBS) (Thermo Fisher Scientific), 5.5 mM glucose, 10,000 U/ml penicillin and 10,000 μg/mL streptomycin sulfate (Thermo Fisher Scientific) under 5% CO_2_.

Primers and antibodies were purchased from: Tocris Bioscience, Bristol, United Kingdom (GSK 650394) and ChemBo Pharma Co., Ltd. (EMD638683) ceramide Ab (Enzo Life Sciences, Inc.). FFAR1 antagonist GW1100/371830 was purchased from Albiochem, EMD Millipore. The activator of protein kinase C 12-*O*-tetradecanoylphorbol-13-acetate (TPA), adenylate cyclase activator forskolin, CPT1 inhibitor etomoxir, and the ceramide synthase inhibitor Fumonisin B1 were purchased from Sigma Aldrich.

Palmitate (sodium palmitate, Sigma Aldrich) / Oleate (Sigma Aldrich) exposure media was supplemented with 2% FBS (ThermoFisher Scientific), 5.5mM glucose (Sigma Aldrich, 10,000 U/ml penicillin and 10,000 μg/mL streptomycin sulfate (Thermofiher Scientific, Invitrogen, Inc.), in addition to 0.44% bovine serum albumin (BSA, fatty acid free) (Roche Diagnostics GmbH, Germany) The fatty acid was incubated with the BSA supplemented medium for a minimum of 40 min at 37 degrees to ensure binding of fatty acid to BSA and cells were washed twice with 2% FBS, 5.5 mM glucose media prior to exposure to Palmitate/Oleate in Palmitate/Oleate exposure media. Palmitate was dissolved in 50% ethanol during heating to 70°C. Control cells were given vehicle with equal amounts of ethanol as the palmitate exposed cells (final concentration of ethanol: 0.12%).

### Caspase-3 activity assay

GLUTag cells were plated (at a density of 250,000 cells/ml) and grown in 6-well plates for 24 h. Cells were then washed twice with low serum medium (2% FBS, 5.5 mM glucose) prior to treatment with 0.125 mM palmitate in the presence or absence of indicated concentrations of Fumonisin B1, GW1100, or Etomoxir in 2% FBS and 5.5 mM glucose for an additional 48 h. Caspase-3 activity assay kit (Cell Signaling Technology, Inc., Danvers, MA) was used according to the manufacturer’s instructions. Briefly, this caspase-3 colorimetric assay is based on the hydrolysis of a substrate by caspase-3, resulting in the release of a fluorescent product. Fluorescence (excitation/emission ~342/441 nm) was measured using appropriate excitation and emission filters or settings.

Fluorescence was normalized against the protein concentration of the individual well (see below).

### Protein assay

GLUTag cells were washed twice with phosphate-buffered saline (PBS) and lysed on ice in a RIPA lysis buffer containing 150 mM NaCl, 20 mM Tris, 0.1% SDS, 1% Triton X-100, 0.25% Na-deoxycholate, 1 mM Na_3_VO_4_, 50 mM NaF, 2 mM EDTA and Protease inhibitor cocktail (Sigma Aldrich) for 30 min. Samples were clarified by centrifugation, supernatants were transferred to new tubes and the total protein concentration was determined with Bio-Rad DC protein assay (method of Lowry [25], using BSA as a standard [Bio-Rad Laboratories, Hercules, CA]).

### Hormone secretion

GLUTag cells were plated at a density of 180,000 cells/ml and grown in 6-well plates for 24–48 h. Cells were then treated with palmitate/oleate at indicated doses for an additional 48 h. Immediately after the 48 h incubation, the medium was discarded and the cells were washed with pre-warmed glucose-free KRBH buffer/0.2% BSA, followed by a 30 min pre-incubation with the same buffer. Cells were then treated for 30min with/without 0.25 mM palmitate in pre-warmed glucose-free KRBH buffer/0.2% BSA. For a subset of experiments, the 30 min palmitate treatment was preceded by a 10min pre-incubation with indicated concentrations of GW1100. Immediately after the incubation with palmitate, the buffer was collected in tubes on ice. GLP-1 content in medium/buffer was analyzed using a total GLP-1 ELISA (Cat. # EZGLP1T-36K, Millipore Corporation) according to the manufacturer's instructions. GLP-1 results were normalized by total protein in the individual wells (see above). All experiments were performed in duplicates and repeated ≥ three times to assess the consistency of results.

### RNA extraction, cDNA synthesis, and quantitative RT-PCR

GLUTag cells were lysed and RNA extracted using Aurum total RNA mini kit (Cat # 7326820) (BioRad Laboratories) according to the manufacturer’s instructions. cDNA was synthesized for qPCR using iScript^™^ cDNA synthesis kit (BioRad Laboratories) according to the manufacturer’s instructions. Glyceraldehyde-3-phosphate dehydrogenase (*GAPDH*) mRNA expression was used as an internal control. A one-step RT-PCR kit with SYBR Green (iScript^™^ one-step RT-PCR kit with SYBR^®^ Green) (BioRad Laboratories) was used for real-time quantitative RT-PCR. This kit utilizes iScript RNase H+ reverse transcriptase and hot-start iTaq DNA polymerase.

For each sample, the mRNA level of each target gene relative to *GAPDH* was estimated by calculating the DeltaCt, or ΔCt (Ct_Target_ Gene− Ct_GAPDH_) and then converting to 2^−ΔCt^. To compare mRNA levels between experimental groups, the ratio of the average 2^−ΔCt^ for each treatment group relative to the control group (2^−ΔΔCt^) was determined for each gene.

Primers were designed using Invitrogen custom primer design software (Invitrogen, Inc). The primer list and specifications are given in S1 Table.

### Western blot analysis

GLUTag cellular protein was extracted using RIPA lysis buffer containing 150 mM NaCl, 20 mM Tris, 0.1% SDS, 1% Triton X-100, 0.25% Na-deoxycholate, 1 mM Na_3_VO_4_, 50 mM NaF, 2 mM EDTA and Protease inhibitor cocktail (Sigma Aldrich) for 30 min on ice. Samples were clarified by centrifugation, the supernatants were transferred to new tubes and the total protein concentration was determined with Bio-Rad DC protein assay using BSA as a standard (Bio-Rad). Equal amounts of protein were then mixed with reducing SDS-PAGE sample buffer, boiled for 5 min and proteins were separated by SDS-PAGE. Samples were electrophoresed on a 10% polyacrylamide gel under denaturing conditions, followed by transfer to PVDF membrane (Bio-Rad Laboratories). Membranes were blocked with 5% milk in PBS-T; primary (over-night at 4°C) and secondary (1 hour at RT) antibody incubations were performed in the same buffer, with three 10-min washes in PBS-T intervening. The anti phospho-p38 was purchased from Abcam, Cambridge, UK (cat# ab195049), and the totp38 and β-actin antibodies were from Santa Cruz, Biotechnology, CA (cat # sc-3533 and sc-47778). Horseradish peroxidase-conjugated secondary antibodies (1:5,000) (Santa Cruz Biotechnology, CA) and ECL (enhanced chemiluminescence) (ThermoFisher Scientific) reagents were used to detect proteins. Images and quantifications were obtained using Molecular Imager ChemiDoc XRS with Quantity One Software v. 4.6.5 (Bio-Rad Laboratories).

### Detection of intracellular reactive oxygen species (ROS)

GLUTag cells were plated at a density of 180,000 cells/ml and grown in 6-well plates for 24–48 h. Cells were then treated with palmitate/oleate as described above at the indicated doses for an additional 6 h or 48 h. Intracellular ROS levels were measured using Image-iT LIVE Green Reactive Oxygen Species Detection Kit (Molecular Probes, Life Technologies Europe BV) as previously described [14] using a fluorogenic marker, 5-(and-6)-carboxy-2′,7′-dichlorodihydrofluorescein diacetate (carboxy-H2DCFDA), that is cleaved in the presence of ROS.

Accordingly, following incubation with indicated concentrations of palmitate/oleate, the cells were then washed with KRBH buffer prior to adding 25 μM carboxy-H2DCFDA to each well. Following 30 minute incubation at 37°C, excess probe was removed by washing the cells again with KRBH buffer. Cells were then lysed in PBS containing 1% Triton X-100. Carboxy-DCF fluorescence in cell lysates was detected at an excitation/emission wavelength of 495/529 nm using a microplate reader (SpectraMax M2, Molecular Devices). Fluorescence was normalized against the protein concentration of the individual well.

### Detection of intracellular ceramide

#### Immunocytochemistry

GLUTag cells were grown on 20 μl/cm^2^ coverslips coated with poly-L-lysine (Sigma Aldrich). Following treatment with 0.125 mM palmitate/oleate in low serum medium (2% FBS, 5.5 mM glucose) for the indicated times, cells were washed with PBS, and 4% paraformaldehyde (Sigma Aldrich) added, followed by incubation with 1% BSA in PBS-T for 30 min to block unspecific binding and an over-night incubation at 4°C with primary ceramide monoclonal antibody (MID 15B4 from Enzo Life Sciences). Cells were washed in PBS and incubated with 2% BSA in PBS for 30 min at room temperature (to avoid non-specific hydrophobic interactions), followed by a 1.5 h incubation at room temperature with secondary anti-mouse ALEXA Fluor 488 Ab (1:500 dilution in PBS + 2% BSA). After washing, cells were incubated with 0.5 μg/ml Hoechst (Sigma Aldrich) for 1 min, washed and mounted on glass slides using Polyvinyl alcohol mounting medium with DABCO^®^, antifading, Sigma Aldrich). Edges of the cover glass were sealed with clear nail polish and slides stored in dark at 4°C pending analysis.

#### Fluorescence measurement

GLUTag cells were grown in 96-well plates. The procedure for immunocytochemistry was followed, and immediately after incubation with the secondary antibody (anti-mouse ALEXA Fluor 488), fluorescence in the wells was measured at an excitation/emission wavelength of 495/519 nm, using a microplate reader (SpectraMax M2, Molecular Devices).

### Propidium iodide staining and flow cytometry

GLUTag cells were plated at a density of 180,000 cells/ml and grown in 6-well plates for 24–48 h. The cells were then cultured for 48 h with or without 0.125 mM palmitate + 0.5% BSA. Cell numbers and viability were determined by incubation with 5 μg/ml propidium iodide for 10 min, followed by trypsinization and flow cytometry analysis using a FacsCalibur instrument (Becton-Dickinson).

### Statistical analyses

Comparisons between multiple groups were made by a one-way ANOVA. Student-Newman-Keul’s *post hoc* test was used. Comparisons between control and single treatment groups were made using two-tailed Student’s *t*-test. *P*<0.05 was deemed statistically significant.

## Results

### Palmitate and oleate exert opposing effects on the formation of reactive oxygen species, activation of the mitogen-activated protein kinase p38 and viability of GLP-1-producing cells

As we and others have previously shown—and analogous to its effect on insulin producing β-cells—palmitate exerts lipotoxic effects and induction of apoptosis in GLP-1-producing cells [17, 26, 27]. This effect is indicated to be aggravated by simulated hyperglycemia, as co-incubation of palmitate with 11 mM glucose slightly but significantly increased caspase-3 activation (18.5±5.95 percent increase) as compared to palmitate alone.

**Fig 1:**
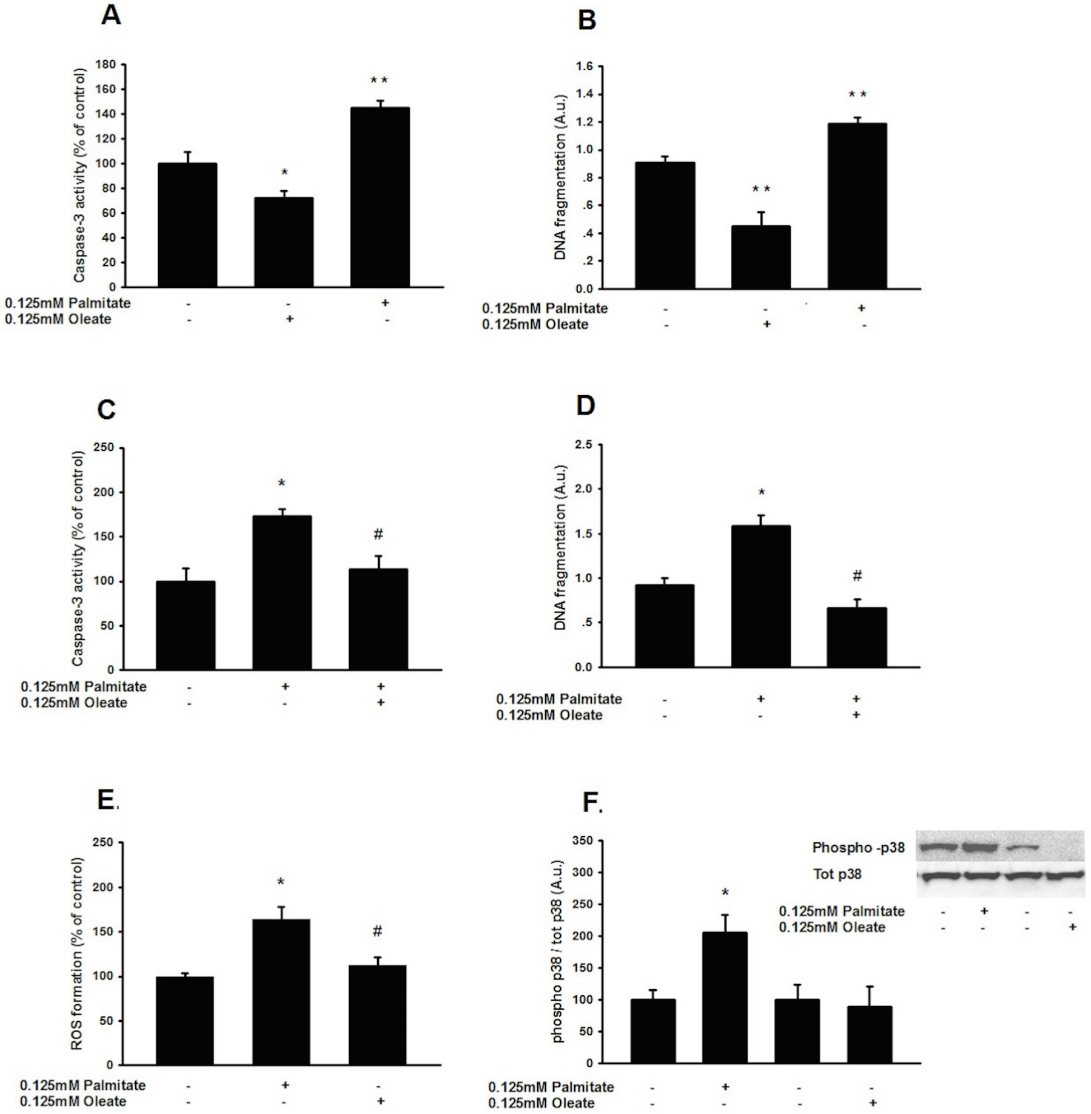
Palmitate and oleate exert opposing effects on the formation of reactive oxygen species, activation of the mitogen-activated protein kinase p38 and viability of GLP-1-producing cells. 0.125 mM oleate significantly decreases—while 0.125 mM palmitate significantly increase—caspase-3 activation (**A**) and DNA fragmentation (**B**) in GLUTag cells following a 48h incubation. Co-incubation of 0.125mM palmitate with 0.125mM Oleate abolishes palmitate induced caspase-3 activity (**C**) and DNA fragmentation (D). ROS production in GLP-1-secreting cells after 48 h (**E**) and phosphorylation of the ROS-sensitive kinase p-38 following 8h (**F**) is increased in response to 0.125mM palmitate, but not in response to 0.125mM Oleate. Bars represent mean ± SEM for n = 3–6 independent experiments analyzed in duplicates. Comparisons between groups were made by a one-way ANOVA, and Student-Newman-Keul’s *post hoc* test. *, *p*<0.05; ***, *p*<0.001 compared with control cells. #, *p*<0.05 compared with palmitate-treated cells.

To determine if the lipotoxic mechanisms are specific to high ambient concentrations of long chain saturated fatty acids, GLUTag cells were exposed to equimolar concentrations of palmitate (16:0) or the monounsaturated fatty acid, oleate (18:1). Our results demonstrate that palmitate increases—while oleate decreases caspase-3 activity (Fig 1A) and DNA fragmentation (Fig 1B) following a 48 h incubation. Furthermore, a co-incubation of palmitate with equimolar concentrations of oleate abolishes palmitate-induced caspase-3 activity (Fig 1C) and DNA fragmentation (Fig 1D).

As we have previously shown that palmitate induced lipotoxicity is mediated, at least in part, by increased production of ROS and activation of the ROS-sensitive mitogen-activated kinase p38 [27], we also evaluated ROS production and p38 phosphorylation in response to oleate. However, neither palmitate induced ROS production, nor activation of the ROS-sensitive kinase p38, is replicated by oleate (Fig 1D and 1E).

### Palmitate-induced lipotoxicity in GLP-1-producing cells is dependent on the formation of ceramide

**Fig 2:**
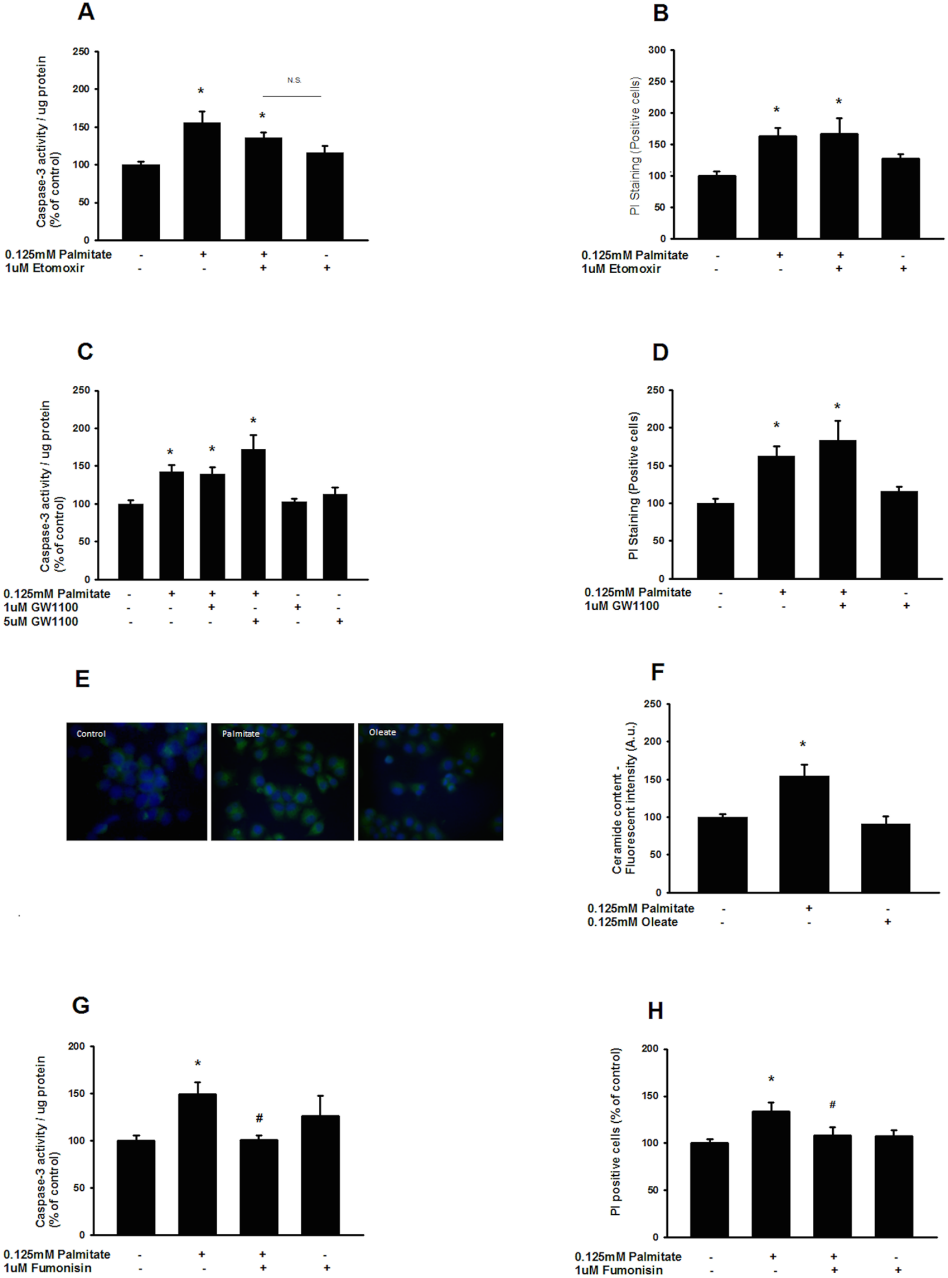
Palmitate-induced lipotoxicity in GLP-1-producing cells is dependent on the formation of ceramide. Co-incubation of 0.125mM palmitate with etomoxir did not significantly alter caspase-3 activation (A) or viability (B) after 48 h. Similarly, co-incubations of 0.125 mM palmitate and FFAR1 antagonist GW1100 did not alter palmitate induced caspase-3 activity (C) or viability (D). GLUTag cells were stained using Hoechst (blue) and a ceramide monoclonal antibody (green) following exposure to 0.125mM palmitate and 0.125mM oleate for 6h (E), where 0.125mM palmitate—but not 0.125mM oleate—increasedthe number of ceramide positive GLUTag cells as assessed by detection of fluorescence proportional to the number of positive cells (F). Co-incubation of 0.125mM palmitate with Fumonisin B1 significantly attenuate palmitate induced caspase-3 activity (G) and reduced viability (H) following 48 h. Bars represent mean ± SEM for n = 3 independent experiment analyzed in duplicates. Comparisons between groups were made by a one-way ANOVA, and Student-Newman-Keul’s *post hoc* test. *, *p*<0.05 compared with control cells. #, *p*<0.05 compared with palmitate-treated cells.

GLUTag caspase-3 activation, and cell viability were assessed following exposure to palmitate in the presence/absence of the carnitine palmitoyltransferase-1 (CPT-1) inhibitor etomoxir, inhibiting mitochondrial fatty acid uptake and thus fatty acid oxidation. Our results demonstrate that co-incubation with etomoxir does not significantly alter caspase-3 activation or viability following a 48 h exposure to palmitate (Fig 2A and 2B). Similarly, co-incubation with GW1100—a reversible antagonist of the palmitate activated FFAR1 receptor—failed to alter the effects of palmitate on caspase-3 activity and viability (Fig 2C and 2D). However, palmitate—but not oleate—leads to a rapid increase of ceramide in GLP-1-producing cells as determined by immunocytochemistry (Fig 2E), as well as fluorescence measurements (Fig 2F). Further, co-incubation with the ceramide synthase inhibitor Fumonisin B1—known to block ceramide formation resulting from both *de novo* synthesis and the salvage pathway of ceramide synthesis—counteracts the induction of caspase-3 activity and reduced viability (Fig 2G and 2H) following a 48 h incubation with palmitate.

### Oleate, but not palmitate, increases the expression of G protein-coupled receptor FFAR1 mRNA and amplifies the acute secretory response of GLP-1 producing cells to fatty acids

To determine the effects of long term exposure to elevated levels of fatty acids on the function of GLP-1-producing cells, we determined the expression of proglucagon, the sodium glucose transporter (SGLT) involved in the secretory response of GLP-1-secreting cells to glucose, and the fatty acid receptors FFAR1 (GPR40) and FFAR3 (GPR43) involved in the response to fatty acids and linked to GLP-1 secretion [28], following exposure to 0.125 mM palmitate/oleate for 6 h, 24 h and 48 h.

**Fig 3:**
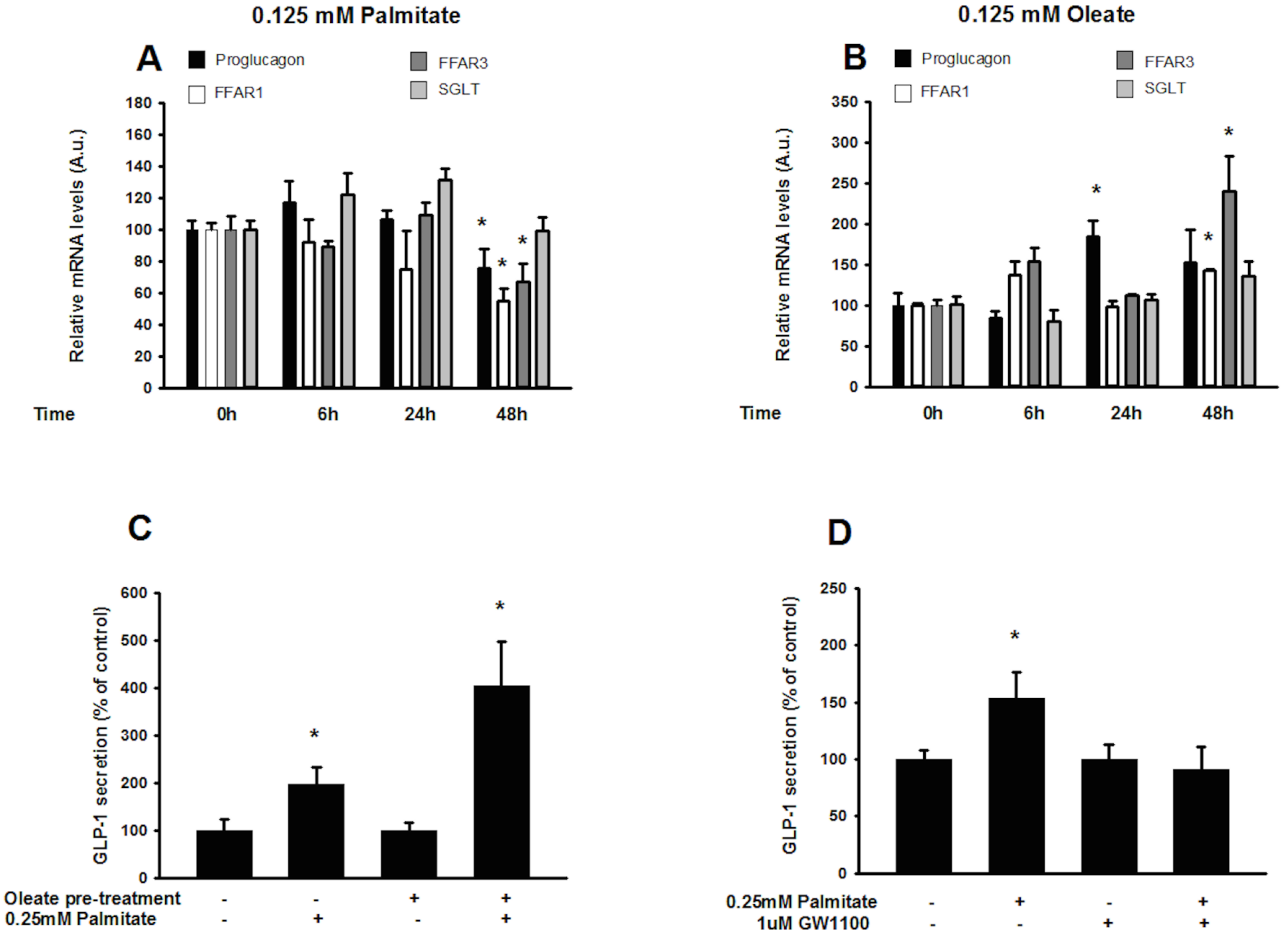
Oleate, but not palmitate, increases the expression of G protein-coupled receptor FFAR1 mRNA and amplifies the acute secretory response of GLP-1 producing cells to fatty acids. Whereas 0.125 mM palmitate significantly reduced GLUTag proglucagon and FFAR1 (GPR40) / FFAR3 (GPR43) mRNA expression after 48h (A), oleate exposure significantly increased the expression of proglucagon / FFAR 1 (GPR40) and FFAR3 (GPR43) mRNA after 24h / 48h respectively (B). GLP-1 secretion in response to 0.5 mM palmitate was increased 2-fold following a 48h exposure to 0.125mM oleate (C). Comparisons between groups were made by a one-way ANOVA, and Student-Newman-Keul’s *post hoc* test. Bars represent mean ± SEM. *, *p*<0.05; ***, *p*<0.001 compared with controls. #, *p*<0.05 compared with palmitate-treated cells.

Our results indicate no significant effects on SGLT expression in response to either fatty acid (Fig 3A and 3B). Further, palmitate downregulates the expression of proglucagon, FFAR 1 (GPR40) and FFAR3 (GPR43) after 48h (Fig 3A), whereas oleate increases the expression of proglucagon after 24h, and of FFAR1 (GPR40) and FFAR3 (GPR43) mRNA following 48h (Fig 3B). Interestingly, GLP-1 secretion in response to 0.5 mM palmitate also increases two-fold in cells cultured in the presence of oleate for 48 h, i.e. palmitate induces a two-fold increase in GLP-1 secretion from untreated cells versus a four-fold increase in secretion from oleate pre-treated cells (Fig 3C). Thissecretory effect of palmitate is indicated, in accordance with what has previously been reported [29, 30], to be mediated by FFAR1 (Fig 3D).

## Discussion

This study demonstrates differential effects of saturated (16:0) and unsaturated (18:1) fatty acids on GLP-1-secreting cell viability and function. The present findings provide novel data in support of a role for ceramide synthesis in palmitate-mediated apoptosis of GLP-1-producing cells. Specifically, our data show that long-term treatment with palmitate increases ceramide production, caspase-3 activity, DNA fragmentation and cell death in GLP-1-producing cells, whereas oleate exerts opposing effects. In addition, oleate—but not palmitate—upregulates FFAR1 (GPR40) / FFAR3 (GPR43) mRNA expression and enhances the subsequent FFAR1-mediated secretory response of GLP-1-producing cells.

In agreement with effects on insulin-producing β-cells, short-term exposure of GLP-1-producing cells to fatty acids has previously been shown to increase GLP-1 secretion without affecting cell viability [26, 31]. Further, lipids are well-recognized stimuli of GLP-1 secretion in humans[32, 33].

However, many of the studies investigating effects of fatty acids on GLP-1 secretion have focused on acute effects, and rendering decreased L-cell mass following a high fat diet, and the reduced GLP-1 plasma levels that have been observed in association with increased BMI, insulin resistance and T2D, it can be hypothesized that toxic effects of chronic hyperlipidemia, *i*.*e*. induction of lipotoxicity, similar to those observed in insulin-producing β-cells both directly and indirectly—through potential reduction in L-cell mass—impairs L-cell secretory capacity, as well as the ability of the surviving L-cells to compensate.

In accordance with the presence of such mechanisms, we have previously shown that—albeit acute exposure stimulates secretion—long term exposure to elevated levels of palmitate induces ROS-dependent lipotoxic effects [26, 27, 34]. The present findings indicate that this lipotoxicity is mediated by ceramide synthesis and specific to the long chain saturated fatty acids (16:0), and thus not replicated by long term exposure to elevated levels of long-chain unsaturated fatty acids (18:1). These findings are in agreement with previously reported differential effects of saturated and unsaturated fatty acids on cell viability [17]. However, herein we demonstrate that this difference is maintained following extended exposure to the fatty acids and we demonstrate increased caspase-3 activity and DNA fragmentation, indicating differences in induction of apoptosis leading to the impaired cell viability. Further, our data suggest that increased β-oxidation is not the main mechanism underlying ROS-mediated lipoapoptosis in GLP-1-producing cells. Although we fail to see a significant effect of palmitate in the presence of CPT-1 inhibition, the increased basal caspase-3 activity under these conditions may mask the effect of palmitate, as there is no significant effect of CPT-1 inhibition on caspase-3 activity or viability following long term palmitate exposure. However, further studies and characterization of fatty acid oxidation in response to saturated (16:0) and unsaturated (18:1) fatty acids in this cell type are necessary to rule out any contribution of increased fatty acid oxidation in palmitate mediated apoptosis. However, the involvement of ceramide synthesis in the induction of apoptosis indicated from the present studies aligns well with what has been reported for other cell types and ROS-mediated lipotoxicity. Specifically, T2D and defective insulin secretion are associated with products generated from saturated fatty acids, whereas unsaturated fatty acids have been shown to exert protective effects. Although the exact mechanisms underlying these observations remain elusive, ceramide synthesis resulting in increased ROS production [35] [36] and oxidative damage [37] is implicated in the induction of lipotoxicity in various cell types. The present study indicates that similar mechanisms may be involved in saturated fatty acid-induced apoptosis of GLP-1-producing cells, whereas long term exposure to elevated levels of unsaturated fatty acids reduces ceramide content and ROS production.

Notwithstanding a rapid turnover of the EECs in the intestine, reports have indicated that L-cell mass can be regulated by external stimuli [12, 13] and the direct impact of fatty acids on GLP-1-producing cell viability demonstrated herein further supports that direct effects of luminal/vascular fatty acids on intestinal L-cells may modulate L-cell mass and thus endogenous GLP-1 secretion from EECs.

We also demonstrate that long term exposure to fatty acids alters the secretory responsiveness of GLP-1 producing cells. Specifically, we demonstrate increased transcription of FFAR1 and enhanced FFAR1-dependent GLP-1 secretion following long term treatment with unsaturated fatty acid (18:1), which may indicate a potential mechanism whereby unsaturated fatty acids exert more potent stimulatory effects on GLP-1-producing cells. However, this is speculative and further studies are needed to clarify if unsaturated fatty acids upregulate functional FFAR1 expression, the role for FFAR1-induced GLP-1 secretion in humans, and the involvement of such potential alterations in the subsequent secretory response of the cells.

The simultaneous up-regulation of both FFAR1 and FFAR3 in response to oleate observed is in line with studies showing the existence of bicistronic mRNA encoding FFAR1 and FFAR3 [38]. Germane to the data presented here, FFAR3 ligands have also been indicated as potential positive regulators of L-cell mass [13].

In conclusion, the present study provides novel data on molecular mechanism underlying differential effects of an unsaturated (18:1) and a saturated (16:0) fatty acids on the induction of apoptosis and lipotoxicity in GLP-1-producing cells. Such effects could potentially contribute to the observations of reduced GLP-1 plasma levels in obese and diabetic patients, indirectly impeding the ability of the β-cell to compensate for insulin resistance through increased insulin secretion. In addition, these effects prompt further assessment of whether dietary fatty acid composition and diets rich in long chain unsaturated fatty acid may be an approach to enhance L-cell mass and GLP-1 secretion. Ultimately, understanding the molecular mechanisms underlying the effects of fatty acids on L-cell mass and function may lead to an increased understanding of the natural unfolding of polygenic T2D, identification of novel signaling pathways, and potential new targets for enhancing endogenous GLP-1 secretion.

## Supporting information

S1 Table Primer list. (PDF)
