# Supplementary material for: Long chain saturated and unsaturated fatty acids exert opposing effects on viability and function of GLP-1-producing cells: Mechanisms of lipotoxicity
Source: PLoS One. 2017 May 16;12(5):e0177605. doi: 10.1371/journal.pone.0177605 (PMC5433723; doi:10.1371/journal.pone.0177605)
Supplement: S1 Table — (PDF) [file pone.0177605.s001.pdf]

Supplemental Table 1 – Primer list.

Name	Sequence	Accession #
GAPDH forward primer	5'-ATGACATCAAGAAGGTGGTG-3'	NM_008084
GAPDH reverse primer	5'-TGTCATACCAGGAAATGAGC-3'	NM_008084
Preproroglucagon forward primer	5'-GATTTTGTGCAGTGGTTGAT-3'	NM_008100.3
Preproroglucagon reverse primer	5'-ACTTCTTCTGGGAAGTCTCG-3'	NM_008100.3
SGLT1 forward primer	5'-TCATGCCAAGACACAGAAGC-3'	AF208031.1
SGLT1 reverse primer	5'-TAGAGGGAGGAATGCAATGG-3'	AF208031.1
GPR41 forward primer	5'-TCCTCAGCACCCTCAACTCT-3'	NM_001033316.2
GPR41 reverse primer	5'-CTAGCTCGGACACTCCTTGG-3'	NM_001033316.2
GPR40 forward primer	5'-GGCCCTATAATGCCTCCAAT-3'	NM_194057.2
GPR40 reverse primer	5'-CCAGGACCTGTTCCCAAGTA-3'	NM_194057.2
